# Self-organisation of complex dynamical systems: from synergetics to neuromorphic systems

**DOI:** 10.3389/fnetp.2026.1736738

**Published:** 2026-04-15

**Authors:** Klaus Mainzer

**Affiliations:** 1 Technical University of Munich (TUM), TUM Senior Excellence Faculty, Munich, Germany; 2 Eberhard Karls University of Tübingen, Carl Friedrich von Weizsäcker Center, Tübingen, Germany; 3 President of the European Academy of Sciences and Arts, Munich, Germany

**Keywords:** hodgkin-huxley circuit, local activity principle, network physiology, neuromorphic computing, nonlinear neuromorphic systems, nonlinear reaction-diffusion systems, synergetics

## Abstract

The intuitive idea of self-organisation in complex dynamical systems is that global patterns and structures emerge from locally interacting elements like atoms in laser beams, molecules in chemical reactions, proteins in cells, cells in organs, neurons in brains, agents in markets, etc. Hermann Haken introduced a mathematically precise and rigorous formalism of synergetics. In this framework we define local activity as the cause of self-organizing complexity which can be tested in an explicit and constructive manner. This principle of local activity can also be defined in the theory of nonlinear electronic circuits. It is not restricted to a certain domain, but can be generalized and proven for the class of nonlinear reaction-diffusion systems in physics, chemistry, biology, and brain research. An example is an improved Hodgkin-Huxley axon circuit model of the brain as network of physiology. It turns out that neuromorphic computing approximates the energetic efficiency of human brains and avoids the enormous increase of energy consumption with traditional digital computing. Obviously, traditional digitalization is closely connected with one of the most challenging problems of mankind–the increasing demand for energy with all its consequences for environmental and climate problems. Thus, synergetics with the local activity principle strongly supports the request for sustainable computing inspired by network physiology.

## Introduction

According to several prominent authors, a main part of 21st century science will be on self-organisation in complex dynamical systems. The intuitive idea is that global patterns and structures emerge from locally interacting elements like atoms in laser beams, molecules in chemical reactions, proteins in cells, cells in organs, neurons in brains, agents in markets etc*.*, by self-organization (Mainzer, 2007a). But what is the cause of self-organization? Complexity phenomena have been reported from many disciplines (e.g. biology, chemistry, ecology, physics, sociology, economy, etc.) and analyzed from various perspectives such as Schrödinger’s order from disorder (Schrödinger, 1948), Prigogine’s dissipative structure (Prigogine, 1980), Langton’s edge of chaos (Langton, 1990) etc. But concepts of complexity are often based on examples or metaphors only. Hermann Haken introduced a mathematically precise and rigorous formalism of synergetics (Haken, 1983). In this framework we define local activity as the cause of self-organizing complexity which can be tested in an explicit and constructive manner.

Boltzmann’s struggle in understanding the physical principles distinguishing between living and non-living matter, Schrödinger’s negative entropy in metabolisms, Turing’s basis of morphogenesis (Turing, 1952), Prigogine’s intuition of the instability of the homogeneous, and Haken’s synergetics are in fact all direct manifestations of a fundamental principle of local activity. It can be considered the complement of the second law of thermodynamics explaining the emergence of order from disorder instead of disorder from order, in a quantitative way, at least for reaction diffusion systems.

The principle of local activity is precisely the missing concept to explain the emergence of complex patterns in a homogeneous medium. This principle can also be defined in the theory of nonlinear electronic circuits (Chua, 1998). It is not restricted to a certain domain, but can be generalized and proven for the class of nonlinear reaction-diffusion systems in physics, chemistry, biology, and brain research. The principle of local activity is the cause of symmetry breaking in homogeneous media.

A completely new application of local activity is the so-called edge of chaos where most complex phenomena emerge. In this particular case, a hidden excitability allows a unit to be destabilized when interacting with dissipative environments. Although a diffusion process has a tendency to equalize differences, an originally dead or inactive cell becomes alive or active upon coupling with other cells by diffusion (Smale, 1974). This phenomenon seems to be counterintuitive, but can mathematically rigorously be proven and confirmed with different applications in reality.

In the parameter spaces of reaction diffusion systems, the domains of local activity can be visualized in computer simulations. The edges of chaos are very small regions with the ability of creation. In an improved Hodgkin-Huxley axon circuit model, the edge-of chaos-domain of the brain can be determined with accuracy. This proves the relevance of these models for network physiology of the brain.

Synergetics and the local activity principle do not only inspire models of the brain, but also brain-orientated (neuromorphic) architectures of computers. It turns out that neuromorphic computing approximates the energetic efficiency of human brains and avoids the enormous increase of energy consumption with traditional digital computing. The main reason of this advantage is the network physiology of the brain. Obviously, traditional digitalization is closely connected with one of the most challenging problem of mankind–the increasing demand for energy with all its consequences for environmental and climate problems. Thus, synergetics with the local activity principle strongly support the request for sustainable computing inspired by network physiology.

## From reaction-diffusion systems to the local activity principle

For the formation of complex biological and chemical patterns, Schrödinger and Prigogine demanded nonlinear dynamics and an energy source as necessary conditions. But, for the exhibition of patterns in an electronic circuit (i.e. non-uniform voltage distributions), the demand for nonlinearity and energy source is too crude. In fact, no patterns can emerge from circuits with cells made of only batteries and nonlinear circuit elements which are not locally active.

In general, a spatially continuous or discrete medium made of identical cells interacting with all cells located within a neighborhood is said to manifest complexity if the homogeneous medium can exhibit a non-homogeneous static or spatio-temporal pattern under homogeneous initial and boundary conditions.

The principle of local activity can be formulated mathematically in an axiomatic way without mentioning any circuit models. Moreover, any proposed unified theory on complexity should not be based on observations from a particular collection of examples and explained in terms that make sense only for a particular discipline, say chemistry. Rather it must be couched in discipline-free concepts, which means mathematics, being the only universal scientific language.

However, in order to keep physical intuition and motivation behind this concept, we start with a special class of spatially-extended dynamical systems, namely the reaction-diffusion equations which are familiar in physics and chemistry. Our first definition of local activity refers to a discretized spatial model which can easily be illustrated by cellular nonlinear networks. All results apply to the associated systems of continuous reaction-diffusion partial differential equations which are analyzed, e.g., in fluid dynamics of physics and chemistry.

Let us consider a spatial lattice of identical cells located at grid points and changing their states by local reaction-diffusion. In general, the change of a local cellular state depends on all the other cellular states in the spatial lattice and (at least in some cases) on the local diffusion of the cell. In mathematical terms, the dynamics of the whole system is defined by a system of discrete reaction-diffusion equations describing the changes of the local cellular states in the spatial lattice (Chua, 1999; Chua, 2005c).

In the discretized as well as continuous case, the state variables pertain to reaction cells lumped at lattice points **r**. This dynamical system implements kinetic equations in chemistry and circuit models in Equation 1 network theories (Mainzer and Chua, 2013). The equations can be represented in the following compact vector form with

$${\dot{V}}_{a}=f_{a}\left(V_{a},V_{b}\right)+{D\nabla }^{2}V_{a}$$ (1)

$${\dot{V}}_{b}=f_{b}\left(V_{a},V_{b}\right)$$ (2)

where

$f_{a}$ ( $V_{a}$ , $V_{b}$ ) $\in R^{m}$ denotes the first *m* components $f_{1}$ ( $V_{a}$ , $V_{b}$ ), $f_{2}$ ( $V_{a}$ , $V_{b}$ ), …, $f_{m}$ ( $V_{a}$ , $V_{b}$ ) of the kinetic term,

$f_{b}$ ( $V_{a}$ , $V_{b}$ ) $\in R^{m}$ denotes the remaining (*n*–*m*) components $f_{m+1}$ ( $V_{a}$ , $V_{b}$ ), $f_{m+2}$ ( $V_{a}$ , $V_{b}$ ), …, $f_{n}$ ( $V_{a}$ , $V_{b}$ ),

**D** denotes an *m* x *m* diagonal matrix defined, and

$\nabla^{2}V_{a}$ $\in R^{m}$ denotes a *m* x 1 vector defined by the *m* discrete Laplacian operators.

The state *variables* $V_{a}$ and $V_{b}$ pertain to only one isolated cell located at the lattice coordinate **r** or to a point **r** ∈ $R^{3}$ in the continuous case. Any dynamical system has some tunable control parameters μ = [ $\mu_{1}\mu_{1}$ … $\mu_{\rho }$ ]^*T*^ associated with changing conditions of the system. Hence, the kinetic term in the reaction-diffusion equation for each cell at location **r** is described by the following cell kinetic (Equations 3, 4):

$${\dot{V}}_{a}=f_{a}(V_{a}\left(r\right),V_{b}\left(r\right);\mu )$$ (3)

$${\dot{V}}_{b}=f_{b}\left(V_{a}\left(r\right),V_{b}\left(r\right);\;\mu \right).$$ (4)

For an *N* x *N* x *N* cubic lattice, there are *N* ^3^ identical cells, each one described by these cell kinetic equations. Since only the first *m* state variables $V_{a}$ (**r**) = [ $V_{1}$ (**r**), $V_{2}$ (**r**), … $V_{m}$ (**r**)]^*T*^ of each cell can interact with the neighboring cells via the diffusion term **D** $\nabla^{2}V_{a}$ (**r**), only the energy and matter associated with the first *m* state variables can flow into a neighbor cell. Henceforth, the state variables $V_{1}$ (**r**), $V_{2}$ (**r**), … $V_{m}$ (**r**) in $V_{a}$ (**r**) are called port variables in analogy with the transfer of goods between islands. The remaining state variables $V_{m+1}$ (**r**), $V_{m+2}$ (**r**), … $V_{n}$ (**r**) in $V_{b}$ (**r**) are called non-port variables. The concept of local activity will be defined in terms of only port variables.

Since the diffusion term can play only a dissipative and hence stabilizing role with $D_{i}$ > 0 in the reaction-diffusion equations, the origin of any complex phenomenon exhibited by these equations can only come from the cell kinetic equations. It can rigorously be proved that if the cell kinetic equations are not locally active for a fixed control parameter μ = $\mu_{0}$ , the reaction-diffusion equations cannot exhibit any complexity regardless of the choices of the diffusion coefficients $D_{i}$ > 0. Moreover, explicit mathematical criteria can be given for testing any cell kinetic equation for local activity. With these criteria, one can identify the active parameter domain 𝒜 of the parameter space μ $\in R^{\rho }$ where a cell kinetic equation is locally active. Since the complement 𝒫 = $R^{\rho }$ \𝒜 can only lead to homogeneous solution of the reaction-diffusion equations, it is called the passive parameter domain.

Since complexity can occur only for parameters located in the active parameter region 𝒜, it follows that local activity is indeed the origin of complexity. A locally-active cell kinetic equation can exhibit complex dynamics such as limit cycles or chaos, even if the cells are uncoupled from each other by setting all diffusion coefficients to zero. It is not surprising that coupling such cells could give rise to complex spatio-temporal phenomena. What is surprising and counter-intuitive is that there exists a proper subset E of the active parameter domain 𝒜, called the edge of chaos where the uncoupled cell kinetic equation is asymptotically stable.

To illustrate this state biologically, a cell is inert or dead in the sense that the concentrations of its enzymes achieved a constant equilibrium. In interaction, however, the cellular system pulses or become alive in the sense that the concentrations of the enzymes in each cell will oscillate indefinitely. By coupling these dead cells via a dissipative diffusion environment, it may be possible for the reaction-diffusion equation to exhibit non-homogeneous patterns and other spatio-temporal phenomena for appropriate diffusion coefficients. The criteria for the edge of chaos correspond mathematically to Prigogine’s instability of the homogeneous and Turing’s properties of instability as origin of morphogenesis.

Since local activity is defined only with respect to *m* port variables in $V_{a}$ = [ $V_{1}$ , $V_{2}$ , … $V_{m}$ ]^*T*^, and since it does not involve the diffusion coefficients $D_{1}$ , $D_{2}$ , …, $D_{m}$ in the *reaction*-diffusion equation, an interaction term $I_{a}$ ≜ **D** $\nabla^{2}V_{a}$ can be defined as port input vector at the diffusion-driven ports. By substituting this input term into the compact vector form of the reaction-diffusion equations, we get the cell kinetic (Equations 5, 6):

$${\dot{V}}_{a}\left(r\right)=f_{a}(V_{a}\left(r\right),V_{b}\left(r\right);\mu )+I_{a}$$ (5)

$${\dot{V}}_{b}\left(r\right)=f_{b}(V_{a}\left(r\right),V_{b}\left(r\right);\mu )$$ (6)

with control parameter μ $\in R^{\rho }$ . They are called forced cell kinetic equations, because $I_{a}$ $\in R^{m}$ can be physically interpreted as an external force applied at the *m* diffusion ports. Since all the cells are identical, this equation represents the state dynamics of an isolated cell driven at its diffusion ports by an external force $I_{a}$ $\in R^{m}$ representing the external world.

The equilibrium states of an isolated cell can be obtained by setting the state change to zero with ${\dot{V}}_{a}$ = **0** and ${\dot{V}}_{b}$ = **0**, namely

$$0=f_{a}\left(V_{a},V_{b};\mu \right)+I_{a}$$ (7)

$$0=f_{b}\left(V_{a},V_{b};\;\mu \right),$$ (8)

and solving these Equations 7, 8 for $V_{a}\in R^{m}$ and $V_{b}\in R^{n-m}$ for each fixed parameter μ $\in R^{\rho }$ . In general, there are multiple equilibrium points for each input $I_{a}$ $\in R^{m}$ .

Let ${\overset{—}{V}}_{a}$ and ${\overset{—}{V}}_{b}$ denote the coordinates of any cell equilibrium point Q , where Q depends on the constant input ${\bar{I}}_{a}$ $\in R^{m}$ and control parameter $\mu \in R^{\rho }$ . In the case of an infinitesimal change $i_{a}$ (*t*) of the constant input $I_{a}$ , we consider infinitesimal deviations $v_{a}$ (*t*) and $v_{b}$ (*t*) in the neighborhood of the equilibrium point Q with coordinates ${\overset{—}{V}}_{a}$ and ${\overset{—}{V}}_{b}$ , namely (Equations 9–11):

$$V_{a}\left(t\right)≜{\bar{V}}_{a}+v_{a}\left(t\right)$$ (9)

$$V_{b}\left(t\right)≜{\bar{V}}_{b}+v_{b}\left(t\right)$$ (10)

$$I_{a}\left(t\right)≜{\bar{I}}_{a}+i_{a}\left(t\right).$$ (11)

In order to approximate the forced cell kinetic dynamics at the cell equilibrium point with constant input $I_{a\;}$ = ${\bar{I}}_{a}$ , we use the Taylor series expansion of $f_{a}$ ( $V_{a}$ , $V_{b}$ ; μ ) and $f_{b}$ ( $V_{a}$ , $V_{b}$ ; μ ) about the cell equilibrium point Q ( $V_{a\;}$ = ${\overset{—}{V}}_{a}$ , $V_{b\;}$ = ${\overset{—}{V}}_{b}$ ). In general, a Taylor series is a representation of a function as an infinite sum of terms that are calculated from the values of the function’s derivatives at a single point. It is usual to approximate a function by using a finite number of terms of its Taylor series. Taylor’s theorem gives quantitative estimates on the error in this approximation. Any finite number of initial terms of the Taylor series of a function is called a Taylor polynomial. The Taylor series of a function is the limit of that function’s Taylor polynomials, provided that the limit exists. If we delete the higher-order terms of the Taylor series, we obtain linearized cell state equations. They can be interpreted as the cell dynamics along a tangent plane at the cell equilibrium point Q which depends on the input $I_{a}$ and the control parameter value μ **.**

We are now able to define local activity at a cell equilibrium point Q . Given any continuous input function of time $i_{a}$ (*t*) for *t* ≥ 0 and assuming zero initial conditions $v_{a}$ (0) = **0**, $v_{b}$ (0) = **0**, a solution of the linearized cell state equations about cell equilibrium point Q is an infinitesimal cell state in the neighborhood of the cell equilibrium point Q , denoted by $v_{a}$ (*t*) and $v_{b}$ (*t*) for *t* ≥ 0. Let us define the local power flow *p*(*t*) ≜ $v_{a}$ (*t*) $\cdot i_{a}\left(t\right)$ as rate of change of energy at time *t* at cell equilibrium point Q ( $V_{a\;}$ = ${\overset{—}{V}}_{a}$ , $V_{b\;}$ = ${\overset{—}{V}}_{b}$ ). Mathematically, the term *p*(*t*) denotes the scalar (dot) product between the two vectors $v_{a}$ (*t*) and $i_{a}$ (*t*).

The principle of local activity is based on the idea that when operating in an infinitesimal neighborhood of a cell equilibrium point Q , a locally-active cell must behave like a unit (e.g., a transistor in technology) operating at an active operating point whereby a small (low-power) input can be converted into a large (high-power) output at the expense of an energy supply (e.g., a battery in the case of a transistor). In general, a cell is said to be locally active at an equilibrium point Q if it is possible to find a local (i.e. infinitesimal) input $i_{a}\left(t\right)$ such that by applying the global input $I_{a\;}$ (*t*) ≜ ${\bar{I}}_{a}$ + $i_{a}$ (*t*), we can extract more infinitesimal energy at Q over some time interval 0 < *T* < ∞ than what the cell has taken from its external environment which consists of the coupling spatial grid of all the other cells.

Let *w*(*t*) be the total energy (i.e. the “infinitesimal sum” or integral of power flow *p*(*t*) ≜ $v_{a}$ (*t*) $\cdot i_{a}\left(t\right)$ accumulated since the initial time *t* = 0 until *t* = *T*. It is convenient though arbitrary to distinguish the reference direction of the total energy entering and leaving the cell at *t* = *T*. If *w*(t) > 0, then there is a net total energy accumulated since the initial time *t* = 0 entering the cell at *t* = *T*. Conversely, if *w*(t) < 0, then at *t* = *T*, the cell is actually delivering energy to the external circuit. In this case, at *t* = *T*, the cell behaves like a local source of energy, rather than a sink.

### Definition of local activity

A cell is said to be locally active at a cell equilibrium point Q if and only if there exists a continuous input time function $i_{a}$ (*t*) $\in R^{m}$ , *t* ≥ 0, such that at some finite time *T*, 0 < *T* < ∞ , there is a net energy flowing out of the cell at *t* = *T*, assuming the cell has zero energy at *t* = 0, namely (Equation 12) (Mainzer and Chua, 2013).

$$w\left(t\right)=\int_{0}^{T}v_{a}\left(t\right)\cdot i_{a}\left(t\right)dt<0,$$ (12)

where $v_{a}$ (*t*) is a solution of the linearized cell state equation about Q with zero initial state $v_{a}$ (0) = **0** and $v_{b}$ (0) = **0**.

### Definition of local passivity

A cell is said to be locally passive at a cell equilibrium point Q if and only if it is not locally active at Q , namely (Equation 13):

$$w\left(t\right)=\int_{0}^{T}v_{a}\left(t\right)\cdot i_{a}\left(t\right)\;dt>0,$$ (13)

for all continuous input time functions $i_{a}$ (*t*) and for all *t* ≥ 0, under zero initial states $v_{a}$ (0) = **0** and $v_{b}$ (0) = **0**.

In the definition of local activity, we need the assumption of zero energy at *t* = 0, because otherwise the cell may have some stored energy *t* = 0 and it could be discharging it to the outside circuit even though it is locally passive. The cell’s ability to act as a source of small-signal energy implies that it can amplify an initially small input signal into a larger-energy signal. The increase in energy must, of course, come from some external energy supply, such as a kind of external pump or battery if the cell is a transistor, or “glucose” if the cell is a neuron. According to the conservation principle of energy, there is nothing coming from nothing, or, in economic terms, there is “no free lunch”.

Mathematically, the signal must be infinitesimally small in order that we can model the cell by only the linear terms in its Taylor series expansion. This in turn allows us to apply well-known linear mathematics and derive explicit analytical criteria for the cell to be locally active at the equilibrium point where the Taylor series expansion is computed. This also proves that complexity originates from infinitesimally small perturbations, notwithstanding the fact that the complete system is typically highly nonlinear.

Intuitively, a cell is locally-active if it is endowed with some excitable “innate” potential, such that under certain conditions, it can become “mathematically alive”, capable of exhibiting oscillation and chaos. The deepest and counter-intuitive property of local activity is that a “mathematically dead” but locally active cell can become explosive, even if it is interfaced with a locally-passive load, or “sink”. That can never happen with a locally-passive cell, whose entropy must increase continuously.

In order to prove that a cell is locally-active at an equilibrium point Q , the definition of local activity requires that an input time function $i_{a}$ (*t*) must be found which initiates a positive energy flow $\int_{0}^{T}v_{a}\left(t\right)\cdot i_{a}\left(t\right)$ *dt* out of the cell at some finite time 0 < *T* < ∞ , assuming $v_{a}$ (0) = **0** and $v_{b}$ (0) = **0**. The definition is intuitively clear and mathematically precise, but misses a constructive procedure whether such an input time function exists or not. Therefore, computationally practical necessary and sufficient conditions must be found to test the local activity of some cell at an equilibrium point.

In natural and engineering sciences, Laplace transforms are used for analysis of linear time-invariant systems (e.g., electrical circuits, harmonic oscillators, optical devices, and mechanical systems). In this analysis, the Laplace transform is sometimes interpreted as a transformation from the time-domain, in which inputs and outputs are functions of time, to the frequency-domain, where the same inputs and outputs are functions of complex angular frequency (in radians per unit time). In any way, given a simple mathematical or functional description of an input or output to a system, the Laplace transform provides an alternative functional description that often simplifies the process of analyzing the behavior of the system, because it converts a system of linear differential equations to a system of linear algebraic equations (Mainzer and Chua, 2013).

Therefore, we consider the Laplace transforms of each component of the vectors $v_{a}$ (*t*), $v_{b}$ (*t*), and $i_{a}$ (*t*) (Equations 14–16):

$${\hat{v}}_{a}\left(s\right)≜\int_{0}^{\infty }v_{a}\left(t\right)e^{-st}dt$$ (14)

$${\hat{v}}_{b}\left(s\right)≜\int_{0}^{\infty }v_{b}{\left(t\right)e}^{-st}dt$$ (15)

$${\hat{i}}_{a}\left(s\right)≜\int_{0}^{\infty }i_{a}\left(t\right)e^{-st}dt\text{ with complex numbers }s=\sigma +i\omega \text{ of the complex domain }C$$ (16)

Applying the Laplace transform to each term of the two linearized cell state equations, we obtain (Equations 17, 18):

$$s{\hat{v}}_{a}\left(s\right)=A_{11}\left(Q\right)\;{\hat{v}}_{a}\left(s\right)+A_{12}\left(Q\right)\;{\hat{v}}_{b}\left(s\right)+{\hat{i}}_{a}\left(s\right)$$ (17)

$$s{\hat{v}}_{b}\left(s\right)=A_{21}\left(Q\right)\;{\hat{v}}_{a}\left(s\right)+A_{22}\left(Q\right)\;{\hat{v}}_{b}\left(s\right).$$ (18)

The solution of the last equation delivers the Equation 19:

$${\hat{v}}_{b}\left(s\right)=(s1–A_{22}{\left.\left(Q\right)\right)}^{–1}A_{21}\left(Q\right)\;{\hat{v}}_{a}\left(s\right)$$ (19)

where the symbol one denotes the identity matrix.

If we substitute this term for ${\hat{v}}_{b}$ (*s*) in the first linearized cell state equation, we obtain the following expression of the input function (Equation 20):

$${\hat{i}}_{a}\left(s\right)=Y_{Q}\left(s\right)\;s{\hat{v}}_{a}\left(s\right),$$ (20)

where $Y_{Q}$ (*s*) ≜ [(s**1** – $A_{11}$ ( Q )) – $A_{12}$ ( Q ) (s**1** – $A_{22}$ ( Q ))^−1^ $A_{21}$ ( Q )] is called the complexity matrix at cell equilibrium Q which promises to deliver the desired computational procedure whether such an input function exists or not.

In the following local activity theorem, the complexity matrix is used as computational procedure to decide whether the local activity property is satisfied for some input function. The concept is at first developed for the scalar case where there is only one port state variable (*m* = 1) so that the complexity matrix $Y_{Q}$ (*s*) is a scalar, henceforth called the complexity function $Y_{Q}$ (*s*). The generalization to the matrix case (*m* > 1) follows the same procedure.

For illustration, the real and imaginary parts of complex number *s* = *a* + *ib* are denoted by Re[*s*] = *a* and Im[*s*] = *b*, in short, *s* = Re[*s*] + *i*Im[*s*]. Then, a positive-real complexity function $Y_{Q}$ (*s*) can be represented with a complex *s-*plane. A complexity function $Y_{Q}$ (*s*) is said to be a positive-real function iff (1) $Y_{Q}$ (*s*) is a real number whenever *s* is a real number, (2) Re [ $Y_{Q}$ (*s*)] ≥ 0 for all *s* with Re[*s*] ≥ 0 where $Y_{Q}$ (*s*) is not singular. Since $Y_{Q}$ (*s*) is assumed to be a rational function, condition (1) is always satisfied. In this case, $Y_{Q}$ (*s*) is a positive-real function iff, the closed right-half *s*-plane is mapped into the closed right-half $Y_{Q}$ -plane.

In a theorem of local activity, it can be proved that a cell with one port state variable is locally active at equilibrium point (Equation 21) Q ( $V_{1\;}$ = ${\overset{—}{V}}_{1}$ ) if, and only if, any one of the following conditions is true (Mainzer and Chua, 2013):

$$\begin{array}{l} \left(i\right)Y_{Q}\left(s\right)\text{has }a\text{ pole in the open right plane Re}\left[s\right]>0\\ \left(\text{ii}\right)Y_{Q}\left(s\right)\text{has }a\text{ multiple pole on the imaginary axis}.\\ \left(\text{iii }\right)Y_{Q}\left(s\right)\text{has }a\text{ simple pole }s=i\omega_{P}\text{ on the imaginary axis and }K_{Q}\left(i\omega_{P}\right)≜\\ \lim_{s\to i\omega_{P}}\left(s-i\omega_{P}\right)Y_{Q}s\text{ is either }a\text{ negative real number},\text{or }a\text{ complex number}\\ \left(\text{iv}\right)\text{Re}\left[Y_{Q}\left(i\omega \right)\right]<0\text{ for some }\omega \in \left(–\infty ,\infty \right). \end{array}$$ (21)

By the same procedure, one can prove the general test for local activity of complexity matrix $Y_{Q}$ (*s*) for any *m* ≥ 2.

The complexity matrix $Y_{Q}$ (*s*) depends not only on the cell equilibrium point Q ( $V_{a\;}$ = ${\overset{—}{V}}_{a}$ ), but also on the cell control parameters μ $\in R^{\rho }$ . For each cell state ( Q , μ ), we can test whether any one of the four conditions in the Local Activity Test is satisfied. This explicit procedure can be derived analytically in simple cases, or numerically by a computer. We can partition therefore the parameter space into a locally-passive domain 𝒫 and a locally-active domain 𝒜, over all possible cell equilibrium points corresponding to $V_{a\;}\in$ $R^{m}$ with 𝒫 ∪ 𝒜 = $R^{m}$ .

By definition, a cell is locally passive iff it is not locally active. Therefore, to prove that local activity is the origin of complexity, it suffices to prove that the reaction-diffusion equation cannot exhibit any form of complexity if the cells are locally passive. But what does complexity mean here?

A spatially continuous or discrete medium made of identical cells which interact with all cells located within a neighborhood (called sphere of influence) with identical interaction laws is said to manifest complexity iff the homogeneous medium can exhibit a non-homogeneous static or spatio-temporal pattern, under homogeneous initial and boundary conditions.

It follows that a reaction-diffusion medium is capable of exhibiting complexity if, and only if, the corresponding continuous reaction-diffusion partial differential equations, or their discretized version, have at least one non-homogeneous static or spatio-temporal solution for some homogeneous initial and boundary conditions. The initial condition is required to be homogeneous since otherwise, we can consider a system made of only cells which are not coupled to each other, such as a system of reactive-diffusion equations with zero diffusion coefficients. This system can exhibit a non-homogeneous static pattern by choosing the initial condition to correspond to any pattern of cell equilibrium states, assuming each cell has two or more equilibrium states.

The main result is that if the cells are strictly locally passive, then all solutions of the reaction-diffusion differential equations must converge to a unique steady state as *t* →∞ . Since the homogeneous steady state consisting of all uncoupled cells at the same equilibrium state is one such solution, it must be the only solution due to the uniqueness property. Therefore, the corresponding medium cannot exhibit any form of complexity.

### Definition of the edge of chaos

An uncoupled cell (with $I_{a}$ = **0**) of a reaction-diffusion equation is said to be on the edge of chaos iff all of its cell equilibrium points are locally active but asymptotically stable. The set E of all locally active parameters μ $\in R^{\rho }$ with this property is called the edge of chaos parameter set (Mainzer and Chua, 2013).

The principle of local activity offers an effective and unified framework for determining whether a dynamical system made of coupled cells can exhibit emerging complex behavior. In general, complexity means non-homogeneous patterns. The principle of local activity is not only a theory, but also a practical and analytical tool determining the regions of local activity, the edge of chaos, and local passivity in a rigorous way. The complex behavior cannot only precisely be predicted, but also completely visualized in computer simulations. Further on, the edge of chaos is a new phenomenon of complexity which was until now not studied. Even though the edge of chaos is a very small domain of local activity and therefore for a long time ignored, it has a crucial role in the emergence of complexity. Their dynamics is analytically represented by differential equations. In order to visualize the emergence of complex patterns in the regions of local activity and at the edge of chaos, the continuous dynamics of the complex systems must be transformed into discrete space versions where visualizations can be computed.

### Examples of local activity and the edge of chaos

The Brusselator model (named after a research group in Brussels) was one of the first systems of equations used to explain self-organizing chemical reactions of the reaction-diffusion type. Based on this model, a theory of dissipative structures operating far from thermodynamic equilibrium was developed by Ilya Prigogine (Prigogine, 1980; Nicolis and Prigogine, 1989). Under stability theory techniques, Prigogine and his group derived a critical bifurcation boundary for the uncoupled cell. They studied stationary and dynamic patterns emerging in the neighborhood of this boundary. But, except for the stability boundaries, the far-from-thermodynamic-equilibrium theory is too coarse to predict sharper and more precise domain of emergent behavior. Especially, they ignored the relatively small subset of the edge of chaos where the emergence of complexity is most likely (Dogaru and Chua, 1998b).

The mathematical model of the Brusselator is defined by two partial differential equations (PDE) with two diffusion coefficients $D_{1}$ and $D_{2}$ and two state variables $V_{1}$ and $V_{2}$ characterizing the chemical dynamics. The cell parameters are denoted by *a* and *b*, the spatial coordinates are denoted by *x* and *y*. The coupling coefficients are assumed as diffusion with $D_{1}$ ≥ 0 and $D_{2}$ ≥ 0. A local activity test can be applied with the four criteria which were proved above.

The complexity matrix can be used to classify each cell parameter point (*a*, *b*) at the equilibrium point $Q_{1}$ with a test algorithm into one of the three disjoint categories:

- Locally Active and Stable: Because there is only one equilibrium point of a Brusselator, this region coincides with the edge of chaos domain. The edge of chaos domain is defined as the region in the cell parameter space where the isolated cell is locally active and stable at least at one equilibrium point.
- Locally Active and Unstable: This region corresponds to the oscillatory or unstable region of an isolated cell.
- Locally Passive: This is the region in the cell parameter space where complex phenomena are unlikely to occur in reaction-diffusion systems.

For cells with only one equilibrium point, every cell parameter point must belong to one of these three disjoint classes. The classification of a cell parameter point into one of these three categories depends on whether there is only one diffusion coefficient, or there are two nonzero diffusion coefficients.

Another example to explain the emergence of complexity with the local activity theory are the Gierer-Meinhardt-equations. On the basis of autocatalysis and lateral inhibition, Gierer and Meinhardt proposed a mathematical model (Gierer and Meinhardt, 1972) to explain pattern formation (morphogenesis) in living systems. Using numerical integration, they were able to produce a number of patterns relevant to the formation of biological structures. An analytical treatment of the Gierer-Meinhardt model from the synergetics perspective (Haken, 1994) was presented in by Haken and Olbrich (Haken and Olbrich, 1978) using the order parameter concept combined with linear stability analysis. While the later approach offers significant contributions in understanding the dynamics of the Gierer-Meinhardt model, it is still too coarse to predict the precise domain in the cell parameter space where emergent behavior may occur. The local activity theory offers a tool for sharpening existing results in the sense that it can identify more precisely those regions in the cell parameter space which are capable of emergent behaviors, and also in fine tuning such regions into a relatively small subset called the edge of chaos where the emergence of complex phenomena is most likely (Dogaru and Chua, 1998).

The edge of chaos is a special case of local activity which has importance, e.g., for the emergence of life or activities of electrical devices in technical networks. In a mathematical sense, it can be proven that initially stable (“dead”) cells become unstable (“alive”) when interacting by a dissipative process. Alan Turing, well-known as logician and mathematician of computability, was one of the first scientists who became aware of this phenomenon. Turing considered a particular case of reaction-diffusion equations (Turing, 1952).

Assume that two identical cells containing two molecules with concentrations $X_{i}$ and $Y_{i}$ , which migrate by diffusion towards each other. The resulting reaction-diffusion equations can be introduced with two port state variables ( $X_{i}$ , $Y_{i}$ ) and positive diffusion coefficients $D_{1}$ and $D_{2}$ (Mainzer and Chua, 2013). Turing’s four two-cell reaction-diffusion equations can be represented into a matrix form. This matrix form is a trivial example of the compact vector form of the reaction-diffusion equations. Since this equation is linear, there is a unique cell equilibrium point for each **I** ∈ $R^{2}$ . The corresponding complexity matrix is denoted by $Y_{Q}$ . The eigenvalues of the matrices ${\;G}_{1}$ and $G_{2}$ in Turing’s two-cell reaction-diffusion equations can easily be calculated. A matrix is said to be stable iff all its eigenvalues have nonpositive real parts. In Turing’s example, both ${\;G}_{1}$ and $G_{2}$ are stable matrices. However the joint matrix ${G=G}_{1}+G_{2}$ after dissipative interaction of the cells is not stable, because one of its eigenvalues is positive. The corresponding complexity matrix $Y_{Q}$ satisfies conditions (i) and (iii) of the local activity test. Therefore, it is locally active.

Since all reaction-diffusion equations presented in Turing’s paper of 1952 are linear, they cannot be used to simulate non-homogeneous spatio-temporal phenomena. To overcome this objection, Stephen Smale considered the same two-cell example but defined the kinetic equation of each cell by a nonlinear vector field in $R^{4}$ (Smale, 1974). He proved rigorously that each uncoupled cell is globally asymptotically stable, and is therefore „dead” in a mathematical sense. But upon coupling the 2 cells by diffusion, he could prove that the resulting reaction-diffusion equations have a global limit-cycle attractor, and hence the cell becomes „alive” in Smale’s mathematical terminology.

This is the special case of local activity which was called the edge of chaos. This phenomenon is counterintuitive, because two (mathematically) „dead” cells interacting by a diffusion process which has a tendency to equalize the cellular concentrations yield a pulsing state. Smale could not solve this problem in a mathematical rigorous form. But, he clearly identified the problem to determine the origin of complexity, namely to axiomatize the properties necessary to bring about oscillation via diffusion. It can now be concluded that the axiomatic properties necessary to bring about oscillation via diffusion is that the uncoupled kinetic cell be on the sharp edge of chaos. In this case, it is asymptotically stable when uncoupled. But its local activity is generated after dissipative interaction in the sense of local activity test condition (iv).

## Synergetics and local activity principle

According to Hermann Haken, synergetics can be defined as the general theory of collective (global) spatial, temporal or functional macrostructures of multi-component (complex) systems. Haken’s view on complexity stems from his studies in laser physics, while Prigogine was inspired by physical chemistry and thermodynamics. In the physical theory of a laser all concepts could be formulated and tested which later were generalized in the conceptual framework of synergetics (Haken, 1983; Weidlich, 2002).

The laser consists of a cavity with parallel mirrors at the two opposite sides and a huge number of more than 10^18^ laser-active atoms. Thus, it is an example of a complex dynamic system of interacting elements. The elements are atoms which can be excited from their ground state to an energetically excited state by some external pumping procedure. This procedure is very interesting in the sense of the local activity principle. According to quantum physics, the atoms make transitions to the ground state by spontaneous emission of photons in random directions. But if the number of excited atomic states passes a certain threshold, the photons propagating back and forth between the mirrors remain longer in the cavity than those in other directions. They interact with the atoms by stimulating the excited ones to emit their photon with the same frequency and wavelength in the same direction. This process initiates an avalanche of photons in macroscopic order in one direction which is known as a laser-beam. Thus, “laser” is an acronym for “light amplification by stimulated emission of radiation”.

Physically, the emergence of a laser-beam is a dynamic phase transition of a complex system surpassing a critical threshold of its control parameter of excitation. Its equations of motion can be derived from the microscopically valid equations of quantum electrodynamics. After the appearance of the macroscopic amplitude of the laser-beam, the laser-active atoms no longer behave randomly and independently but instead exhibit a well-ordered and cooperative light emission pattern determined by the laser-light mode.

According to synergetics, the laser-light mode has become an order parameter “slaving” the dynamic behaviour of the other atoms of the system. In this case, the atoms organize themselves according to the global pattern determined by certain order parameters. The collective self-organization of the atoms is a synergetic effect caused by the critical phase transition of the whole system. Obviously, this phase transition means an immense simplification of mathematical modeling: Instead of dealing with more than more than 10^18^ equations for all atoms and all photons it is sufficient to use equations for a few order parameters characterizing a few laser-active atomic variables of the laser-light mode. The dynamics of the other variables is fully determined (“slaved”) by the order parameters. In the sense of the local activity principle, order parameters correspond to a local activity of a few laser-active atoms enslaving the whole system and exhibiting new patterns and structures of matter.

The special case of a laser system can be generalized as mathematical formalism applicable to a broad class of physical and nonphysical systems (Haken, 1993; Weidlich, 2002). According to statistical mechanics, we distinguish the microscopic level of microstates of the elements of a complex system (e.g., states of atomic movements in a fluid) from the macroscopic level of the macrostate of the whole system (e.g., aggregate state of ice) depending on certain control parameters (e.g., external temperature). The microstates at time *t* are $q_{1}$ (*t*), $q_{2}$ (*t*), …, $q_{n}$ (*t*). The vector **q**(t) = [ $q_{1}$ (*t*), $q_{2}$ (*t*), …, $q_{n}$ (*t*)] describes the state of the system on the microscopic level. The change of states **q**(t) is determined by a set of known microscopic dynamical (Equation 22):

$$\frac{q_{i}}{dt}=N_{i}\left(q;\alpha \right)+F_{i}\left(t\right)\text{ with }i=1,2,\ldots ,n,\text{or in compact vector form}\frac{dq}{dt}=N\left(q;\alpha \right)+F\left(t\right).$$ (22)

The set of functions *N*_*i*_ represents nonlinear functions of the state variables **q** and of external control parameters α . The set $F_{i}$ (*t*) represents small stochastic forces describing additional exogenous accidental effects on the system which can be ignored.

The dynamics of the system is analyzed in the neighborhood of an instability. Therefore, we start with a known solution $q_{00}$ of the microscopic dynamical equations for given constant control parameters $\alpha_{0}$ . In the simplest case $q_{00}$ is a constant vector representing the stable solution of **N**( $q_{00}$ ; $\alpha_{0}$ ) = 0. If the value of the external control parameter is now shifted from $\alpha_{0}$ to α , the system variable gets the form (Equation 23):

$$q\left(t\right)=q_{0}+w\left(t\right)$$ (23)

with the (stable or unstable) stationary solution $q_{0}$ of **N**( $q_{0}$ ; $\alpha_{0}$ ) = 0 and the small deviation **w**(*t*) from this stationary solution.

The question arises whether $q_{0}$ remains a stable solution or whether there evolves another dynamics after the shift $\alpha_{0}$ → α . Therefore, **q**(t) = $q_{0}$ + **w**(*t*) is inserted into the microscopic dynamical equations (with **F**(*t*) = 0). In a next step, the equation is expanded into a Taylor series with respect to **w**(*t*), obtaining

$\frac{{dw}_{i}}{dt}$ = $\sum_{j=1}^{n}L_{ij}$ ( $q_{0}$ , α ) $w_{j}$ + $M_{i}$ ( $q_{0}$ , α ; **w**) with *i* = 1, 2, … , *n*, or in compact vector form (Equation 24):

$$\frac{dw}{dt}=L\left(q_{0},\alpha \right)w+M\left(q_{0},\alpha ;w\right).$$ (24)

The first term with Equation 25:

$$L=\left(\left(L_{ij}\right)\right)=\left(\left(\frac{\partial N_{i}}{\partial q_{j}}|\begin{array}{c} q=q_{0}\; \end{array}\right)\right)$$ (25)

contains the linear terms in **w** in the Taylor expansion and the second term contains the higher expansion terms. The quadratic and cubic expansion terms (Equation 26), for example, have the components

$$M_{i}\left(q_{0},\alpha ;w\right)=\sum_{a,b}M_{i,ab}^{\left(2\right)}w_{a}w_{b}+\sum_{a,b,c}M_{i,abc}^{\left(3\right)}w_{a}w_{b}w_{c}+\ldots$$ (26)

For small deviations **w**(*t*) the higher terms can be neglected, in order to analyze the approximate linearized (Equation 27):

$$\frac{dw}{dt}=L\left(q_{0},\alpha \right)w.$$ (27)

Its solutions **w**(*t*) can be decomposed into eigensolutions $w^{\left(k\right)}$ (*t*) with Equation 28:

$$w\left(t\right)=\sum_{k}c_{k}w^{\left(k\right)}\left(t\right)\text{ and}w^{\left(k\right)}\left(t\right)=\exp\left(\lambda^{\left(k\right)}t\right)v^{\left(k\right)}$$ (28)

The eigenvalues $\lambda^{\left(k\right)}$ and the eigenvectors $v^{\left(k\right)}$ with their adjoint eigenvectors ${\tilde{v}}^{\left(k\right)+}$ have to satisfy the Equations 29, 30:

$$L\left(q_{0},\alpha \right)\;v^{\left(k\right)}=\lambda^{\left(k\right)}v^{\left(k\right)}\text{ with components }\sum_{j=1}^{n}L_{ij}v_{j}^{\left(k\right)}=\lambda^{\left(k\right)}v_{i}^{\left(k\right)}\text{ and}$$ (29)

$${\tilde{v}}^{\left(l\right)+}\;L\left(q_{0},\alpha \right)=\lambda^{\left(l\right)}{\tilde{v}}^{\left(l\right)+}\text{ with components }\sum_{i=1}^{n}{\tilde{v}}_{i}^{\left(l\right)*}L_{ij}=\lambda^{\left(l\right)}{\tilde{v}}_{j}^{\left(l\right)*}$$ (30)

These eigenvectors $v^{\left(k\right)}$ and the adjoint eigenvectors ${\tilde{v}}^{\left(l\right)+}$ form a bi-orthogonal system.

$\left({\tilde{v}}^{\left(l\right)}|{\tilde{v}}^{\left(k\right)}\right)$ = $\delta_{lk}$ with respect to the scalar product and the conjugate complex $u_{j}^{*}$ of $u_{j}$ in:

$$\left(u|v\right)=\sum_{j=1}^{n}u_{j}^{*}\cdot v_{j}=u^{+}\cdot v\;.$$

How can the stability of the solution **q**(*t*) = $q_{0}$ + **w**(*t*) be guaranteed after the shift $\alpha_{0}$ → α of the external control parameter of the system? Mathematically, the stability depends on the eigenvalues $\lambda^{\left(k\right)}$ which are, in general, complex numbers $\lambda^{\left(k\right)}$ = ${\lambda^{\prime }}^{\left(k\right)}$ + *i* ${\lambda^{\prime \prime }}^{\left(k\right)}$ . In the following, one must distinguish the cases of negative and positive real parts of the eigenvalues with Equation 31:

$$\text{Re}\left(\lambda^{\left(s\right)}\right)={\lambda^{\prime }}^{\left(s\right)}<0\text{ and Re}\left(\lambda^{\left(u\right)}\right)={\lambda^{\prime }}^{\left(u\right)}>0$$ (31)

If only eigenvalues $\lambda^{\left(s\right)}$ with negative real parts exist, the corresponding deviations $w^{\left(s\right)}$ (*t*) = exp( $\lambda^{\left(s\right)}$ *t*) $v^{\left(s\right)}$ remain small with time so that the approximate linearized equation is sufficient for all times and the system remains stable in the neighborhood of $q_{0}$ after the shift $\alpha_{0}$ → α of the external control parameter of the system.

If, instead, a few eigenvalues $\lambda^{\left(u\right)}$ with positive real parts exist, the corresponding deviations $w^{\left(u\right)}$ (*t*) = exp( $\lambda^{\left(u\right)}$ *t*) $v^{\left(u\right)}$ grow exponentially with time so that the approximate linearized equation becomes invalid. In this case, the stationary solution becomes unstable. Therefore, the expansion of the deviation **w**(*t*) must be substituted by Equation 32:

$$w\left(t\right)=\sum_{u}\xi_{u}\left(t\right)\;v^{\left(u\right)}+\sum_{s}\xi_{s}\left(t\right)\;v^{\left(s\right)}$$ (32)

with amplitudes $\xi_{u}\left(t\right)$ for the unstable modes and $\xi_{s}\left(t\right)$ for the stable modes. These amplitudes are determined by inserting them into the exact nonlinear equations of motion with the complete Taylor expansion. The corresponding equations can be transformed into equations of the amplitudes $\xi_{u}\left(t\right)$ and $\xi_{s}\left(t\right)$ by taking the scalar products with the adjoint eigenvectors ${\tilde{v}}^{\left(u\right)}$ and ${\tilde{v}}^{\left(s\right)}$ in Equations 33, 34:

$$\frac{d}{dt}\left({\tilde{v}}^{\left(u\right)}|w\left(t\right)\right)=\left({\tilde{v}}^{\left(u\right)}|\text{Lw}\left(t\right)\right)+\left({\tilde{v}}^{\left(u\right)}|M\left(w\left(t\right)\right)\right)\;\text{and}$$ (33)

$$\frac{d}{dt}\left({\tilde{v}}^{\left(s\right)}|w\left(t\right)\right)=\left({\tilde{v}}^{\left(s\right)}|\text{Lw}\left(t\right)\right)+\left({\tilde{v}}^{\left(s\right)}|M\left(w\left(t\right)\right)\right)$$ (34)

The application of the eigenvector equations and the bi-orthogonality relation yield (Equations 35, 36):

$$\frac{d\xi_{u}\left(t\right)}{dt}=\lambda^{\left(u\right)}\xi_{u}\left(t\right)+M_{u}\left(\xi_{u},\xi_{s}\;\right)\;and$$ (35)

$$\frac{d\xi_{s}\left(t\right)}{dt}=\lambda^{\left(u\right)}\xi_{s}\left(t\right)+M_{s}\left(\xi_{u},\xi_{s}\right).$$ (36)

The expressions $M_{u}$ and $M_{s}$ can be determined in $\left({\tilde{v}}^{\left(u\right)}|M\left(w\left(t\right)\right)\right)$ and $\left({\tilde{v}}^{\left(s\right)}|M\left(w\left(t\right)\right)\right)$ by inserting the decomposition of **w**(*t*) in unstable and stable modes into **M**(**w**). The quadratic and cubic terms of the Taylor expansion yield bilinear and trilinear expressions in $\xi_{u},$ $\xi_{s}$ of the form (Equations 37, 38):

$$M_{u}(\xi_{u},\xi_{s})=\sum_{r,r^{\prime }}m_{u,rr^{\prime }}^{\left(2\right)}\xi_{r}\xi_{r^{\prime }}+\sum_{r,r^{\prime },r^{\prime \prime }}m_{u,rr^{\prime }r^{\prime \prime }}^{\left(3\right)}\xi_{r}\xi_{r^{\prime }}\xi_{r^{\prime \prime }}\;\text{and}$$ (37)

$$M_{s}(\xi_{u},\xi_{s})=\sum_{r,r^{\prime }}m_{s,rr^{\prime }}^{\left(2\right)}\xi_{r}\xi_{r^{\prime }}+\sum_{r,r^{\prime },r^{\prime \prime }}m_{s,rr^{\prime }r^{\prime \prime }}^{\left(3\right)}\xi_{r}\xi_{r^{\prime }}\xi_{r^{\prime \prime }}$$ (38)

with sums over *r*, *r*’, *r*’’ extending over all stable and unstable modes.

The coupled nonlinear equations for the amplitudes $\xi_{u}\left(t\right)$ and $\xi_{s}\left(t\right)$ of the unstable and stable modes are equivalent to the exact microscopic equations of the whole dynamical system (for **F**(*t*) = 0). They allow us to prove a different dynamic behavior of $\xi_{u}\left(t\right)$ and $\xi_{s}\left(t\right)$ due to the different kind of eigenvalues $\lambda^{\left(u\right)}$ and $\lambda^{\left(s\right)}$ .

In many complex dynamical systems including the laser and diffusion-reaction systems, there exist for a given control parameter only very few eigenvalues $\lambda^{\left(u\right)}$ with a small but positive real part whereas most eigenvalues $\lambda^{\left(s\right)}$ have large negative real parts. According to the coupled nonlinear equations for the amplitudes, the $\xi_{u}\left(t\right)$ begin to increase exponentially whereas the $\xi_{s}\left(t\right)$ decrease exponentially. But the nonlinear terms of the equations modify this initial behavior. In the further evolution the $\xi_{s}\left(t\right)$ adapt their behavior to the $\xi_{u}\left(t\right)$ which grow to macroscopic order. Therefore, the amplitudes $\xi_{u}\left(t\right)$ are distinguished as order parameters of the whole system. The adaptive behavior of the $\xi_{s}\left(t\right)$ is in a first approximation be described by a so-called adiabatic elimination, i.e. by neglecting the time derivative of $\xi_{s}\left(t\right)$ and yielding the approximate (Equation 39):

$$\xi_{s}\approx –\frac{1}{\lambda^{\left(s\right)}}M_{s}\left(\xi_{u},\xi_{s}\right).$$ (39)

These equations can be used to express the stable amplitudes $\xi_{s}$ in terms of the unstable amplitudes $\xi_{u}$ in an equation $\xi_{s}$ = $f_{s}$ ( $\xi_{u}$ ) for all $\xi_{s}$ . In the sense of synergetics, the stable amplitudes are slaved by the few dominant order parameters of the unstable amplitudes.

Synergetics provides a comprehensive modeling for the emergence of order in a far-from-thermodynamic-equilibrium dynamics. There are various other approaches explaining the emergence of order in different fields of application, such as chemistry, biology, electrical engineering, and even sociodynamics (e.g. Prigogine, Gierer-Meinhard, Weidlich) which were mentioned above. In principle, they can be reconstructed using the formalism of Haken’s synergetics. However, the methods best suited to specific tasks depend on the particularities of the respective field of application. The local activity approach emphasises not only the distinction between stable and unstable states, but also between locally passive and locally active states. Stable and locally active cells play a particularly important role because, after dissipative interaction, they can generate new structures, orders and chaos (edge of chaos). Special algorithms (Local Activity Test) have been proposed for this purpose. As will be shown later, these methods prove to be particularly suitable for calculating the activation of technical cells (e.g. memristors, transistors).

Overall, the Local Activity Approach can be summarised as follows:

The Local Activity approach relates to dynamical systems defined by partial differential equations (PDE) (e.g., reaction-diffusion equations, cellular nonlinear networks) with diffusion coefficients and state variables characterizing the dynamics. The elements („cells“) of the system are characterized by cell parameters and spatial coordinates. The coupling coefficients of cells are assumed as diffusion. A local activity test can be applied with the four criteria (21) (i) - (iv) which were mentioned above.

A complexity matrix can be used to classify each cell parameter point at the equilibrium point with a test algorithm into one of three disjoint categories:

- Locally Active and Stable: This region coincides with the edge of chaos domain. The edge of chaos domain is defined as the region in the cell parameter space where the isolated cell is locally active and stable at least at one equilibrium point.
- Locally Active and Unstable: This region corresponds to the oscillatory or unstable region of an isolated cell.
- Locally Passive: This is the region in the cell parameter space where complex phenomena are unlikely to occur in reaction-diffusion systems.

These different domains can be distinguished at different cell parameter points in computer simulations (Mainzer and Chua, 2013).

## From local activity principle to memristors

On the horizon of future chip technology is the vision of neuromorphic computers, mimicking the human brain with billions of neurons and synaptic connections. A technical unit modeling a living neuron with synapses needs features of memories, digital circuits, and a form of analog information processing. A strong candidate fulfilling all these requirements is the memristor, a circuit element, which was theoretically suggested more than 50 years ago (Chua, 1971). Modern technology assumes that the memristor will bring a new wave of innovation in electronics, packing more bits into smaller volumes and equipped with a non-volatile memory preventing the loss of data. Its brain-oriented architecture will also avoid the „von Neumann bottleneck” of traditional digital computing with increasing consumption of energy.

The memristor device has generated immense interests among both device researchers and the memory-chip industry alike (Strukov et al., 2008). These interests were due to the high potential economic impact of the HP (Hewlett-Packard) breakthrough. Since the titanium-dioxide HP memristor could be scaled down to about 1 nm and is compatible with current IC technology, many industry experts are predicting that nano memristor devices would eventually replace both flash memories and DRAMS (Dynamic Random Access Memory). Indeed, a PC with no booting time, and which remembers all data prior to unplugging the power, could become standard features.

Memristor is an abbreviation for “memory resistor” und was predicted as the fourth missing circuit element with respect to the basic equations of electric circuits (Hayes, 2011). These equations are defined for the four quantities voltage (*v*), current (*i*), charge (*q*), and magnetic flux ( φ ). Each equation determines a relation between two of these variables. The simplest relation between voltage and current is Ohm’s law *v* = *Ri*, meaning that voltage is proportional to current. The constant of proportionality is given by the resistance *R*. If a current of *I* amperes flows through a resistance of *R* Ω, then the voltage with respect to the resistance is *v* volts. Geometrically, the graph of current versus voltage for a resistor is a straight line with slope *R*.

There are six possible pairings of four variables. The two pairs (*v*, φ ) and (*i*, *q*) are already related by definition *v* = $\frac{d\varphi }{dt}$ and *i* = $\frac{dq}{dt}$ . The three pairs (*v*, *i*), ( φ , *i*), and (*q*, *v*) define a resistor, inductor, and capacitor, respectively. The missing equation relating charge *q* and magnetic flux φ is called memristor. Obviously, the memristor was found by theoretical arguments of symmetry and completeness.

There are two classes of memristors, namely, locally-passive memristors and locally-active memristors. The conventional resistor, capacitor, inductor, and the locally-passive memristor are passive circuit elements, which must be distinguished from active devices, such as transistors, which can amplify signals and inject power into circuits. However, locally-active memristors are active devices and hence can also amplify signal, with a power supply, just like transistors. The memristor is a nonlinear device defined by the graph of a curve in the flux vs. charge plane, whose slope, called the memristance, varies from one point to another (Chua, 2011).

A transistor is a three-terminal device with three connections to a circuit. It acts as a switch or amplifier, with a voltage applied to one terminal controlling a current flowing between the other two terminals. Although a locally-active memristor has only two terminals, it can also realize these functions. On the other hand, locally-passive memristors can be used to build both memory and digital logic without the need for a power supply. This is why they are called non-volatile memories. Metaphorically speaking, the memristor has a built-in sense of history. A signal applied at one moment can affect another signal that travels the same path later. The first signal realizes this control by setting the internal state of the memristor to high or low resistance.

Therefore, in a neuromorphic computer, locally-passive memristors would not totally supplant a transistor, but supplement them in memory functions and locic circuits. Memristors could play the role of synapses. In biological neural networks, each nerve cell communicates with other cells through thousands of synapses. An important mechanism of learning is realized by adjusting the strength of the synaptic connections. In an artificial neural network, synapses must be small, but effective structures. Locally-passive memristors satisfy all the needed requirements. They change their resistance in response to the currents that flow through them. This operation suggests a direct way of modeling the adjustment of synaptic strength.

Recall that there are two qualitively distinct kinds of memristors, namely locally passive memristors and locally active memristors. The HP memristor is locally passive because it does not require a power supply, and is said to be non-volatile. The potassium and sodium ion channels in the classic Hodgkin-Huxley nerve membrane circuit model can be considered locally-active memristors, powered by a sodium and a potassium pump whose energy derives from ATP molecules. In contrast, synapses are locally passive memristors capable of retaining their synaptic efficacies over long periods of time without consuming any power.

Since our brains process information using only synapses and axons, it follows that circuits made of both types of memristors should be able to emulate higher brain functions as well. The long-term potentiation (LTP) phenomenon associated with long-term memory can also be emulated by a memristor. Many associative memory phenomena, such as the Pavlovian dog behavior, can be emulated by a memristor circuit. If brains are made of memristors, then we can expect that electronic circuits made of both locally passive and locally active memristors may someday emulate human minds (Mullins, 2009). The key to this fundamental process is to uncover how local activity could lead to the emergence of complex patterns from a mass of homogeneous brain tissues. In neurons and memristors, the local activity principle is not only a formal model, but biological and technical reality.

At the centre are the Hodgkin-Huxley equations, which were used for the first time to calculate the action potentials of nerve signals in the brain (Hodgkin et al., 1949; Hodgkin and Huxley, 1952a; Hodgkin and Huxley, 1952b; Hodgkin and Huxley, 1952c; Hodgkin and Huxley, 1952d). This makes it possible to introduce cellular neuronal networks that are defined by reaction-diffusion equations. Mathematically, the brain proves to be a complex dynamic system whose dynamics can be modelled according to these laws. In a next step, this neurobiological model is translated into an electrotechnical model with electrotechnical circuits. Central to this is the principle of local activity of these circuits to explain the action potentials and the new electrotechnical unit of a memristor, with which neurobiological synapses and neurons can be represented electrotechnically. Memristive circuits form the basis of neuromorphic systems with which real and analogue computing can be realised.

### Memristive neuromorphic computers and network physiology

The architecture of electronic neuromorphic systems is brain-oriented and can be used to implement real and analogue computing (Mainzer, 2024b). Similar to quantum technology and quantum computers, the hardware of neuromorphic systems is currently undergoing dynamic development with different approaches. It is orientated to network physiology. The human organism is an integrated network, where complex physiological systems, each with its own regulatory mechanism, continuously interact to coordinate their functions. Coordinated network interactions among organs are essential to generating distinct physiological states (Ivanov, 2021). Physiological interactions occur at multiple levels of integration and across spatiotemporal scales to optimize organ functions and synchronize their dynamics. These interactions are mediated by various signaling pathways that work in parallel to facilitate stochastic and nonlinear feedbacks across scales leading to different coupling forms.

All cells in the human body possess specific electrical properties crucial for their functions. The understanding of this phenomenon is supported by computer simulations of a mathematical model, which are validated by experimental data. For example, heart dynamics can be quantified using difference logistic equations, and it correlates with improved overall survival rates in cancer patients (Costa et al., 2025).

Concerning technical brain modeling, memristive neuromorphic systems that use memristors to model synaptic and neuronal signaling processes are central. As in the development of digital computing, it is important that the hardware is supplemented by software based on it, in which the programming of neuromorphic systems can be realised. There are various hardware materials that are currently being used to realise memristive computer architectures. A key advantage of neuromorphic systems is the energy efficiency of these network architectures, similar to the human brain, compared to the enormous energy consumption of digital von Neumann architectures in digital computing.

Instead of biological materials, the device level or circuit level components of neuromorphic computing are realized by a variety of new materials on nano-scaling. Mathematically, the memristor is defined with a function $R\left(q\right)$ (“memristance function”), in which the change of the magnetic flux Φ with the charge q is being held (Equation 40), i.e.

$$R\left(q\right)=\frac{d\Phi \left(q\right)}{dq}.$$ (40)

The temporal change of the charge q defines the current $i\left(t\right)$ with Equation 41:

$$i\left(t\right)=\frac{dq}{dt}.$$ (41)

The temporal change of the magnetic flux Φ defines the voltage $v\left(t\right)$ with Equation 42:

$$v\left(t\right)=\frac{d\Phi }{dt}$$ (42)

The result is that the voltage v at a memristor about the current i depends directly on the memristance (Equation 43):

$$v=R\left(q\right)i$$ (43)

This is reminiscent of Ohm’s law v=Ri which defines that the voltage v is proportional to current *i* with the resistance R as a proportionality constant. However, the memristance is not constant, but depends on the state of the charge. Conversely, for electricity, it holds (Equation 44):

$$i=G\left(q\right)v,$$ (44)

where the function $G\left(q\right)=$ ${R\left(q\right)}^{-1}$ is called “memductance” (composed of the English word “memory” for memory and “conductance” for conductivity).

A memristor can be generalized as a memristive system. A memristive system is no longer reduced to a single state variable and a linear charge- or flow-driven equation.

A memristive system is an arbitrary physical system, which is defined by a set of internal state variables $\vec{s}$ (as a vector). It follows a general input-output (Equation 45):

$$\vec{y}\left(t\right)=g\left(\vec{s},\vec{u},t\right)\vec{u}\left(t\right)$$ (45)

with the input $\vec{u}\left(t\right)$ (z. B. Voltage) and the output $\vec{y}$ (e.g., electricity). The development of a state is generally determined by a differential (Equation 46):

$$\frac{d\vec{s}}{dt}=f\left(\vec{s},\vec{u},t\right)$$ (46)

Memristive systems exhibit exceptionally complex and non-linear behavior. Typical is the hysteresis curve in a v/i -coordinate system.It runs in closed loops through the pinched hysteresis loop. In general, hysteresis refers to the behavior of the output variable of a system that reacts to an input variable with a delayed (Greek hysteros) signal and varies. The behavior does not only depend directly on the input variable, but also on the previous state of the output variable. If the input variable is the same, the system can adopt one of several possible states.

As “neuristors”, memristive systems simulate the behavior of synapses and therefore become interesting for neuromorphic computers. For this purpose, the ion channels in a circuit model are regarded as memristive systems. Hodgkin-Huxley’s time-dependent conductivity $G_{k}$ of the potassium ion channel is replaced by a charge-controlled memristor dependent on a state variable. Hodgkin-Huxley’s time-dependent conductivity $G_{Na}$ of the sodium ion channel is replaced by a charge-controlled memristor which depends on two state variables. These well defined quantities explain precisely the empirical measurement and observation data of synapses and neurons (Sah et al., 2014).

But how can such neuristors be technically realized with appropriate materials? R. Stanley Williams of the company Hewlett-Packard (Silicon Valley) constructed a version for the first time in 2007, which has meanwhile been constantly simplified and improved (Williams, 2008). Imagine a crossbar network of intersecting vertical and horizontal wires, reminiscent of a wire mesh. The intersections of a vertical and horizontal wire are connected by a switch. To close the switch, a positive voltage is applied to both wires. To open it, the charge is reversed.

In order to achieve memrestive behavior, the switches are constructed according to a specific architecture. It is reminiscent of a sandwich in which a titanium dioxide layer a few nanometers thick lies between two platinum electrodes (as “slices of bread”). The lower titanium dioxide layer serves as an insulator. The upper titanium dioxide layer has oxygen deficiencies. One can imagine them as small bubbles in a beer - with the difference that they cannot escape. This titanium oxide layer has a high conductivity. If a positive voltage is applied, the oxygen deficiencies shift. This reduces the thickness of the lower insulation layer and increases the overall conductivity of the switch. A negative charge, on the other hand, attracts the positively charged oxygen deficiencies. This increases the insulation layer and reduces the overall conductivity of the switch.

The memristive behavior becomes apparent when the voltage is switched positively or negatively: Then the small bubbles of the oxygen deficiencies do not change, but remain where they are. The border between the two titanium dioxide layers is “frozen”, so the switch can “remember” how much voltage was last applied. It works like a memristor.

Other memristors use silicon dioxide layers a few nanometers in size, which require only low costs. The crossbar memories manufactured by Hewlett-Packard already have an enormous packing density of approx. 100 Gibit/ ${cm}^{2}$ . They could also be combined with other semiconductor structures. Therefore, it cannot be excluded that they initiate the development of neuromorphic structures to simulate the human brain.

There are many possibilities to realize memristors with appropriate materials. They are often based on transition meta-oxides (TMOs). Different metal oxides can generate different numbers and types of resistance states which determine the weight values stored on the memristor. Among a variety of other materials, chalcogenide memristors have also been used to simulate the behavior of synapses. They enable ultra-fast switching speeds at nanosecond scale. Polymer-based memristors have low cost and tunable performance. Ferroelectric materials have been applied for analog memory for synaptic weights and devices. Graphene is used to construct more compact circuits. Carbon nanotubes are used to realize dendrites on neurons. They have the advantage to produce the scale of neuromorphic systems with number of neurons and synapses as well the density of synapses. They can also interact with living tissues or used in prosthetic applications.

Further on, silicon-based synaptic transistors and oxide-based synaptic transistors are used for neuromorphic implementations. Neuromorphic components such as synapses are also fabricated by organic electrochemical transistors and nanoparticle transistors. Organic transistors have the advantage of low-cost processing and flexibility such as organic memristors. In general, material science plays an important role to produce smaller, faster, and more efficient neuromorphic devices. It is a challenge of future research to understand the different implications of new materials for the functionality of neuromorphic computing (Nirmal et al., 2024).

Memristor crossbar architectures research and development is focussed on memristive devices (Xiao et al., 2024). Memristive devices are an emerging technology that has the potential to revolutionise traditional memory and computing concepts (Liu and Zeng, 2022). They are characterised by their ability to store and process information in the form of resistance changes. These properties are extremely attractive for the development of novel memory and neuromorphic computing applications. The basic structure of a single memristor is quite simple. In these components, the active layer is located between two metal electrodes. This is referred to as a MIM (metal-insulator-metal) structure (Schuman et al., 2017).

As an alternative to the hardware of neuromorphic systems on an electronic (memristive) basis, neuromorphic systems are already being developed on a photonic basis (Farmakidis et al., 2024). Instead of electrons, photons, i.e. light, are now used to transmit information. This technology is still in its infancy, but promises enormous advantages over electrotechnical circuits in terms of energy and the data speed of information transmission. The development of new nanomaterials goes hand in hand with future computing technologies. A standardisation of the various photonic technologies will be a necessary prerequisite for the industrial production and application of photonic neuromorphic chips. Photons can also be understood as quantum systems and thus open up a link to quantum computing. In the end, photonic neuromorphic quantum systems, quasi artificial “brains of light”, are emerging as a future hardware of artificial intelligence (Sun et al., 2021; Mainzer, 2024a).

## Future perspectives and requests

Against this background, this article is also a plea for the integration of synergetic principles in hardware and software engineering (e.g., the concepts of order parameters and local activity). Only those who know these principles can use the advantages of self-organisation for computing and AI technologies. Equally, however, only those who know the advantages and disadvantages of the various hardware materials will ultimately be able to realise neuromorphic chips. Neuromorphic systems will be a key technology for an energetically sustainable and responsible AI on the background of network physiology.

Besides these future AI application, it is inspiring to remember that the search for an explanation of self-organisation has deep historical and philosophical roots. In the 19th century, on the background of Darwin’s theory of evolution, the Aristotelian concept of entelechy was criticized as an old-fashioned teleology contradicting causal explanations of modern science. Nevertheless, until the beginning of the 20th century, biologists of vitalism argued that many of the basic problems of biology cannot be solved by a philosophy in which organisms are considered as “machines”. Therefore, the vitalists (e.g., the German biologist Hans Driescher) strongly believed in a particular “vital force” which could not be reduced to mechanistic concepts. Entelechy was interpreted as a purposive and organizing field directing the growth of organisms.

In his book “Creative Evolution” (1907), the French philosopher Henri Bergson introduced an “élan vital”, which should explain the self-organization and spontaneous morphogenesis of things in an increasingly complex manner (Bergson, 1911). “Élan vital” was translated in the English edition as “vital impetus”, but usually it means “vital force”. It is a literary explanation for evolution and development of organisms, which Bergson linked closely with consciousness. Bergson’s philosophical concept was sometimes misunderstood as a particular force which could be embedded into an inanimate substance and activated like electricity, inspired by another metaphorical term of Bergson, the “current of life”. Therefore, Bergson’s “élan vital” is no scientific term in a strict sense of natural sciences which can be empirically tested, confirmed or refuted.

Nevertheless, it became a paradigm at the turn to the 20th century inspiring many scientists and philosophers until today. A fundamental problem in physical, biological, and physiological systems is to understand phenomena where global behaviors across systems emerge out of networked interactions among dynamically changing entities with coupling forms that are often nonlinear and change as a function of time. In addition to the state of individual organ systems, coordinated network interactions among systems and sub-systems are essential to generate distinct physiologic states and behaviors at the organism level, such as wake and sleep stages, cognition, consciousness and unconsciousness (Ivanov, 2021). Thus, with respect to the new research field of network physiology, Bergson’s link of evolution with the emergence of consciousness seems to be less speculative. With the local activity principle and synergetics, we have (mathematically) rigorous explanations of this phenomenon on the background of network physiology.
